# A Randomized Clinical Study Investigating the Stain Removal Efficacy of Two Experimental Dentifrices

**DOI:** 10.1111/jerd.13373

**Published:** 2025-01-13

**Authors:** C. R. Parkinson, G. R. Burnett, G. Smith, M. Pradhan, J. Gallob, J. Qaqish

**Affiliations:** ^1^ Haleon Weybridge Surrey UK; ^2^ Haleon Warren New Jersey USA; ^3^ All Sum Research Center Ltd. Mississauga Ontario Canada

**Keywords:** abrasivity, alumina, aluminum oxide, chemical cleaning, dentifrice, extrinsic dental stain, sodium tripolyphosphate

## Abstract

**Objective:**

This study aims to evaluate extrinsic tooth stain removal and whitening efficacy of two experimental dentifrices containing (i) 5% sodium tripolyphosphate (STP)/1% micronized alumina or (ii) 5% STP/1% micronized alumina with abrasive silica (ED2) compared to a regular fluoride dentifrice (RFD) following 8 weeks of use.

**Materials and Methods:**

This was a single‐center, randomized, controlled, blind, three‐arm, stratified, parallel‐group study. Eligible participants underwent clinical assessment of stain on the facial/lingual surfaces of maxillary and mandibular teeth using the modified Lobene stain index (MLSI), and shade of the facial surfaces of the central and lateral maxillary incisors using the VITA Bleachedguide 3D‐Master (VITA) shade guide. Participants brushed twice daily with their assigned dentifrice and returned to the clinic for clinical assessments at weeks 4 and 8.

**Results:**

A total of 272 participants completed the study (281 screened, 279 randomized). Both experimental dentifrices demonstrated statistically significant reduction from baseline and significant differences compared to RFD for MLSI and VITA shade scores at 4 and 8 weeks. 10 treatment‐related treatment‐emergent adverse events were reported in seven participants. All were resolved at the end of the study.

**Conclusions:**

This study demonstrated that dentifrices containing 5% STP/1% micronized alumina provide enhanced stain removal and shade improvements compared to a regular dentifrice. Study treatments were generally well tolerated.

**Clinical Significance:**

A low‐abrasion daily‐use fluoride dentifrice containing 1% micronized alumina and 5% STP, suitable for people at risk of erosive toothwear and/or dentin hypersensitivity, can provide clinically meaningful superior reductions in tooth stain, which translates into a visibly noticeable improvement in tooth shade.

## 1 Introduction

The appearance of teeth, particularly tooth color, is an important concern for many people with as many as 70% of adults reported to be dissatisfied with the color of their teeth [1]. Previous research has reported that tooth color has an impact on social perceptions [2], and the potential to impair psychological well‐being [3].

One of the primary functions of a dentifrice is to clean teeth and help control the build‐up of extrinsic dental stains. Extrinsic dental stain results from discoloration of the acquired salivary pellicle on the surface of the tooth and dental plaque through adsorption of chromogens from the diet, smoking, and some medications to proteinaceous components of the salivary pellicle and/or plaque [4]. Poor oral hygiene resulting in inadequate removal of plaque (and salivary pellicle) may also influence the accumulation of dental stains. Generally, extrinsic dental stain can be reduced by twice‐daily use of a dentifrice, however, over time the level of dental stain can build up, especially if there is inadequate brushing technique or exposure to a particularly chromogenic diet [4, 5].

Dentifrices help control the rate of accumulation of dental stains by employing dental‐grade abrasives and/or by the addition of chemically active cleaning or bleaching compounds to a dentifrice formulation [6]. Dental‐grade abrasives function by physically removing stains from tooth surfaces during toothbrushing, typically with the most effective stain removal provided by the more abrasive compounds [5, 6, 7, 8]. However, these higher abrasive compounds are intrinsically linked to higher wear of enamel and dentin [6, 7, 8]. A range of physical parameters have been shown to affect stain removal and wear properties of abrasives including particle hardness, shape, size, distribution, and concentration [5, 8, 9]. In the development of dentifrices, consideration must be given to balancing the desired stain‐removal efficacy with the need to minimize dental wear.

Dentifrices containing alumina (aluminum oxide) are highly effective at removing stains, but they often lead to dentifrices with higher abrasivity. Relative dentin abrasivity (RDA) is an accepted standardized measure used to quantify the abrasiveness of a dentifrice formulation on dentin [10]. Dentifrices with RDA values ≤ 250 are considered suitable for daily use; however, the use of much lower abrasivity dentifrices may be more appropriate for people at risk of erosive toothwear or those with dentin hypersensitivity to limit further wear of enamel and/or exposed dentin [11].

In addition to abrasives, chemical cleaning agents can also be added to dentifrices to complement the physical removal ability of abrasives [6]. Certain chemical cleaning agents can help prevent the deposition of extrinsic stains by interfering with the binding process of chromogens to plaque or the acquired pellicle. Polyphosphates such as sodium tripolyphosphate (STP) have been shown to modify the structure of the salivary pellicle and the binding of chromogens to the pellicle thereby facilitating the removal of protein‐bound stain during tooth brushing, and inhibiting stain accumulation post‐tooth brushing [12].

Recently, it has been reported that micronized alumina abrasive (ca. 5 μm particle size), formulated at 1% in a dentifrice, can provide effective stain control with low relative dentin abrasivity (RDA ~ 40) [13, 14, 15], and exploratory stain‐removal clinical studies have reported that the combination of 1% micronized alumina and 5% STP to be highly effective at removing and preventing dental stain compared to a dentifrice containing silica abrasive alone [14, 15]. However, no studies have reported on the tooth‐whitening (tooth color) efficacy of these low‐moderate abrasion formulations as measured by tooth shade assessment. Toothpaste can be effective at stain removal; however, this stain‐removal activity may not necessarily translate to meaningful (consumer‐noticeable) improvements in tooth whiteness. This study sought to understand if the reported stain removal and stain prevention properties of low abrasion 5% STP and 1% micronized alumina toothpaste [14, 15] translate into meaningful improvements in tooth whiteness (as measured by tooth shade).

The objective of this study was to evaluate the extrinsic tooth stain‐removal efficacy of two experimental (antisensitivity) dentifrices containing the stain‐removal system (i) 5% STP and 1% micronized alumina/5% KNO_3_ (RDA 40) and (ii) 5% STP and 1% micronized alumina with standard dental‐grade abrasive silica/5% KNO_3_ (RDA 120) compared to a regular fluoride dentifrice after 4 and 8 weeks of twice‐daily brushing, as assessed by MLSI and examiner assessment against the VITA Bleachedguide 3D Master shade guide. The null hypothesis tested was that there was no difference between baseline and Week 8 stain removal as measured by MSLI.

## 2 Materials and Methods

### 2.1 Study Design

This was a single‐center, 8‐week, randomized, controlled, blinded, three‐treatment, parallel‐design, stratified study in healthy volunteers with clinically confirmed extrinsic dental stains (originating from diet and/or smoking) on the surfaces of their anterior teeth. The study was designed to investigate changes in tooth stain and shade, following the use of two experimental dentifrices (ED1 and ED2) twice daily for 4 and 8 weeks compared to a regular fluoride dentifrice (included as a reference dentifrice, representative of a regular commercially available dentifrice). The study was conducted at a clinical site: All Sum Research Center Ltd., Florida, USA. The study protocol and documentation were reviewed and approved by an independent ethics committee (Veritas IRB, Tracking # 2022–3081–11,688‐3), and the study was conducted in accordance with Good Clinical Practice. Subjects were recruited by the clinical site, primarily from their local volunteer database. The study was registered at ClinicalTrials.gov (NCT05459558) prior to commencement of the study.

At screening (Visit 1, Day 0), subjects gave their written informed consent to participate in the study. Demography, relevant medical history, and current medications/treatments were recorded, followed by oral soft tissue (OST) and oral hard tissue (OHT) examinations. Subjects meeting the relevant study criteria were considered eligible to proceed with baseline assessments on the same day. At baseline (Visit 2, Day 0), subjects refrained from oral hygiene procedures for at least 6 h, underwent clinical assessment of the intensity (I) and extent (A) of stain on the facial surfaces of their six maxillary anterior teeth and six mandibular anterior teeth (tooth numbers 6–11 and 22–27) and the lingual surfaces of their six mandibular anterior teeth (tooth numbers 22–27) using the MacPherson modification [16] of the Lobene Stain Index [17] (MLSI). The shade of the facial surfaces of the central and lateral maxillary incisors (tooth numbers 7–10) were assessed clinically using the VITA Bleachedguide 3D‐MASTER (VITA Zahnfabrik, H. Rauter GmbH & Co. KG, Germany) [18]. Subjects who fulfilled all inclusion/exclusion criteria were enrolled in the study.

Qualifying subjects with a total MLSI (A × I) of ≥ 15 for the facial surfaces of the 12 anterior teeth and a mean tooth shade of ≥ 11 on the facial surfaces of the 4 maxillary incisors and those who met all other entry criteria were stratified according to their baseline total MLSI (A × I) (low) < 45; high ≥ 45 and smoking status (smoker; nonsmoker) and were randomized to one of three study treatments.

- Experimental dentifrice 1 (ED1): 5% w/w STP, 1% w/w micronized alumina (and 5% KNO_3_, 1100 ppm fluoride as sodium fluoride (NaF)).
- Experimental dentifrice 2 (ED2): 5% w/w STP, 1% w/w micronized alumina, 2% dental‐grade silica (and 5% KNO_3_, 1100 ppm fluoride as sodium fluoride (NaF)).
- Reference fluoride dentifrice (RFD): 1100 ppm fluoride as NaF (Aquafresh Cavity Protection, USA Marketed).

Both experimental dentifrices contained 5% KNO_3_ as they are indicated to provide relief from the pain of dentin hypersensitivity.

Randomized subjects brushed for two‐timed minutes, twice daily (morning and evening) with their assigned study dentifrice for the next 8 weeks and recorded each brushing in the diary provided; significant changes in diet or smoking status were also noted in the diary. Subjects, having refrained from oral hygiene procedures for at least 6 h, returned to the clinic after 4 and 8 weeks of treatment (Visits 3 and 4, respectively) for clinical assessments of dental stain (MLSI) and tooth shade (VITA) in the same way and by the same examiner as at baseline.

Blinding: The examiner, study statistician, data‐management staff, and other employees of the sponsor were blinded to treatment. All dentifrices were overwrapped to mask their identity and facilitate blinding.

An OST examination was carried out prior to the stain/shade assessments at each visit to evaluate soft tissue abnormalities.

### 2.2 Subjects

Inclusion/Exclusion criteria: Inclusion criteria consisted of consented, subjects 18–65 years old, of either sex and in good general health, with no known or suspected allergy or intolerance to the study materials or ingredients, and willingness to comply with scheduled visits, product usage requirements, study procedures, and lifestyle restrictions (from screening onward, subjects were not be permitted to use chewing gum, use chewing tobacco, or to smoke, vape, or use tobacco products). At screening, subjects were required to have: ≥ 16 natural teeth, including at least 11 of the 12 anterior teeth, with the facial surfaces of all anterior teeth (maxillary and mandibular) and lingual surfaces of anterior teeth (mandibular only) gradable for MLSI; with the presence of extrinsic dental stain which originated from the diet and/or use of tobacco products.

At baseline, eligible subjects were required to have a total MLSI (A × I) ≥ 15 for the facial surfaces of the 12 anterior teeth and a mean tooth shade ≥ 11 on the facial surfaces of the 4 maxillary incisors, and who met all other entry criteria.

General exclusion criteria included the presence of chronic disease that could affect study outcomes; pregnancy or breastfeeding; participation in another clinical study or receipt of an investigational drug within 30 days of the screening visit. General oral exclusions included: dental prophylaxis within 8 weeks; gross periodontal disease or treatment of periodontal disease within 12 months; scaling/root planing within 3 months; use of professionally dispensed or over‐the‐counter bleaching/whitening products (excluding daily‐use whitening dentifrices) within 3 months; and use of mouthwashes or medication containing ingredients considered to cause dental staining. Specific dentition exclusions included teeth with current or recent caries (treatment < 12 months before screening); teeth with exposed dentin but with deep, defective, or facial restorations; teeth used as abutments for fixed or removable partial dentures and those with full crowns, orthodontic bands, or cracked enamel; teeth with surface irregularities; discoloration of nonvital teeth due to trauma, tetracycline stain, restorations, and hypo‐ or hyperplasic areas, which would have prevented consistent grading. Other exclusions included subjects who were employees of the investigational site (either directly or indirectly involved), subjects with a known or suspected intolerance to the study products, and subjects with OST examination findings at screening which, in the opinion of the investigator could interfere with the conduct of the study.

### 2.3 Procedures and Assessment Criteria

To standardize the assessment conditions, MLSI and VITA assessments were performed by direct observation by the same trained and calibrated examiner throughout the study, in the same room with consistent light levels. Before the assessment, the examiner could brush or floss the teeth, as required, to remove any impacted food. Teeth were air‐dried before and as required during the examination.

MLSI: Extrinsic tooth stain was measured using an established clinical measure of dental stain—the MLSI [16]. The extrinsic dental stain was assessed on the facial surfaces of the six maxillary and six mandibular anterior teeth (tooth number 6–11 and 22–27), and the lingual surfaces of the six mandibular anterior teeth (22–27) at baseline, Week 4, and Week 8 (Visits 2–4). The facial and lingual surfaces of each assessable tooth were divided into four regions (gingival, distal, body, and mesial). The “gingival” region was defined as a crescent‐shaped band, approximately 2 mm wide, adjacent to the free margin of the gingiva and extending to the crest of the interdental papillae of the adjacent teeth. The remainder of both the facial and lingual tooth surfaces was then subdivided into three regions: distal, body, and mesial.

The intensity (I) and area (A) of the stain were assessed for each region of the facial and lingual surfaces of each assessable incisor and canine tooth. The intensity of stain covering each region was scored using a four‐point scale (0 = no stain; 1 = light stain; 2 = moderate stain; 3 = heavy stain). The area of stain covering each region was also scored using a four‐point scale (0 = no stain detected, only tooth color; 1 = stain covering up to one‐third of the tooth surface; 2 = stain covering between one‐third and two‐thirds of the tooth surface; 3 = stain covering more than two‐thirds of the tooth).

The mean total MLSI (A × I) score for each subject at each visit was calculated as the mean of all MLSI (A × I) scores over all non‐missing gingival, body, mesial, and distal sites on the facial surfaces of the six maxillary anterior teeth and six mandibular anterior teeth, and the lingual surfaces of the six mandibular anterior teeth.

VITA: Tooth shade of the facial surfaces of the 4 central and lateral maxillary incisors (tooth numbers 7–10) were assessed by a single, trained, clinical examiner using the VITA Bleachedguide 3D‐MASTER (VITA Zahnfabrik, H. Rauter GmbH & Co. KG, Germany), which utilized a value‐ranked ordered scale from 1 (the lightest) to 29 (the darkest). The shade level of each tooth surface was scored visually by the clinical examiner with reference to the VITA Bleachedguide 3D‐MASTER, consisting of 27 individual shades [18].

The mean VITA shade score for each subject at each visit was calculated as the mean VITA shade score over all non‐missing facial surfaces of the four central and lateral maxillary incisors.

### 2.4 Examiner Repeatability

Throughout the study, the examiner conducted repeat MLSI and VITA tooth shade assessments (with a minimum of 10 min and a maximum of 60 min between repeat assessments for a given subject). 10 subjects were planned to be randomly selected for repeat MLSI and VITA tooth shade assessments at each assessment time point (Visits 2, 3, and 4); a total of 30 repeat assessments (for both MLSI and VITA tooth shade assessments) over the duration of the study. The scores of the initial assessment were not visible to the examiner or scribe when the repeat assessment was carried out. The repeat dental assessments were compared to the respective original assessments and were used to investigate intra‐examiner variability. The repeat assessments were not used in any efficacy analyses. A weighted kappa (*κ*) coefficient and 95% confidence interval (CI) were calculated to assess intra‐examiner repeatability for both area and intensity assessments. Repeatability was categorized as excellent (*κ* > 0.75), fair to good (*κ* ≥ 0.4 and ≤ 0.75), or poor (*κ* < 0.4) [19].

### 2.5 OST Examination

The OST examination was accomplished by direct observation and palpation with retraction aids as appropriate.

### 2.6 Safety

All examiner‐observed and subject‐reported adverse events (AEs) and OST abnormalities were recorded.

### 2.7 Statistical Analysis

It was planned to screen a sufficient number of subjects to randomize approximately 300 subjects (approximately 100 per arm) to ensure approximately 270 subjects (approximately 90 per arm) completed the study. The study was powered sufficiently (> 90%) to provide evidence of the superiority of an Experimental Dentifrice compared to the reference dentifrice for both stain removal (MLSI) and whitening (VITA shade) after 8 weeks under the graphical approach method [20] (with an initial two‐sided alpha allocation of 5% for testing within each Experimental Dentifrice) to adjust for multiple comparisons and preserve a two‐sided 10% family‐wise error rate. The simulation showed that under the graphical approach methodology [20] and the above underlying assumptions for an Experimental Dentifrice, the power to show statistical significance in all four comparisons for that Experimental Dentifrice (reduction from baseline and superior reduction compared to the reference dentifrice in both mean total MLSI [A × I] and mean VITA shade score) was > 90%. Approximately 100 subjects per group (approximately 300 in total) were intended to be randomized to account for a drop‐out rate of up to 10% prior to Week 8.

The primary analysis population was the modified intent‐to‐treat (mITT) population, defined as those subjects who received study treatment and had at least one post‐baseline efficacy measurement. The primary endpoint for this study was the change from baseline in the mean overall MLSI (A × I) at Week 8 within each study product. The secondary efficacy endpoints were the change from the baseline and between‐treatment comparison in the mean VITA shade score and MLSI at Weeks 4 and 8. Hypothesis testing of the change from baseline in the mean overall MLSI (A × I) and mean VITA shade score at Week 8 for each Experimental Dentifrice employed the graphical approach [20] to maintain an overall 2‐sided family‐wise error rate at 5% within each Experimental Dentifrice (10% overall). The *p* values for the primary efficacy endpoint and secondary efficacy endpoints: change from baseline in mean overall MLSI [A × I] and VITA score within product change for each Experimental Dentifrice and subsequent comparisons with the reference dentifrice at Week 8) were adjusted depending on results of the other endpoints and the completion of the graphical approach testing. For all other secondary and exploratory endpoints, there were no adjustments for multiplicity, and unadjusted *p* values were presented.

The change from the baseline was analyzed using a mixed model with repeated measures (MMRM). For MLSI‐related endpoints, fixed effects were included for smoking status, study product, visit, and study product × visit interaction. The respective baseline mean MLSI score was included as a covariate. For the VITA shade‐related endpoints, fixed effects were included for smoking status, baseline overall MLSI (A × I) stratification (< 45 or ≥ 45), study product, visit, and study product × visit interaction. The respective baseline mean VITA shade score was included as a covariate. In each MMRM, subjects were included as repeated measures with an unstructured covariance matrix. The change from baseline at a given timepoint was evaluated within each study product and between study products based on the least square means (using observed margins) and differences between least square means respectively at the given timepoint with the Kenward Rogers degrees of freedom approach applied. The assumptions of normality and homogeneity of variance in each MMRM were planned to be investigated and if found to be in potential violation of these assumptions, the van Elteren test, adjusted for the randomization stratification, was performed to support the MMRM results.

For both MLSI and VITA scores, the assumption of normality and homogeneity were potentially in violation, and the nonparametric test (the Van Elteren test, adjusted for the randomization stratification) was performed for each comparison.

AEs and OST abnormalities were reported using descriptive statistics. All analyses were performed using SAS version 9.4 (SAS Institute Inc. Cary, NC, USA).

## Availability of Data and Materials

The full trial protocol is available at: ClinicalTrials.gov; study number NCT06150573. Anonymized individual participant data and study documents can be requested for further research from https://haleon‐study‐register.idea‐point.com/StudyRegister.aspx.

## 3 Results

### 3.1 Demographics and Baseline Characteristics

**FIGURE 1:**
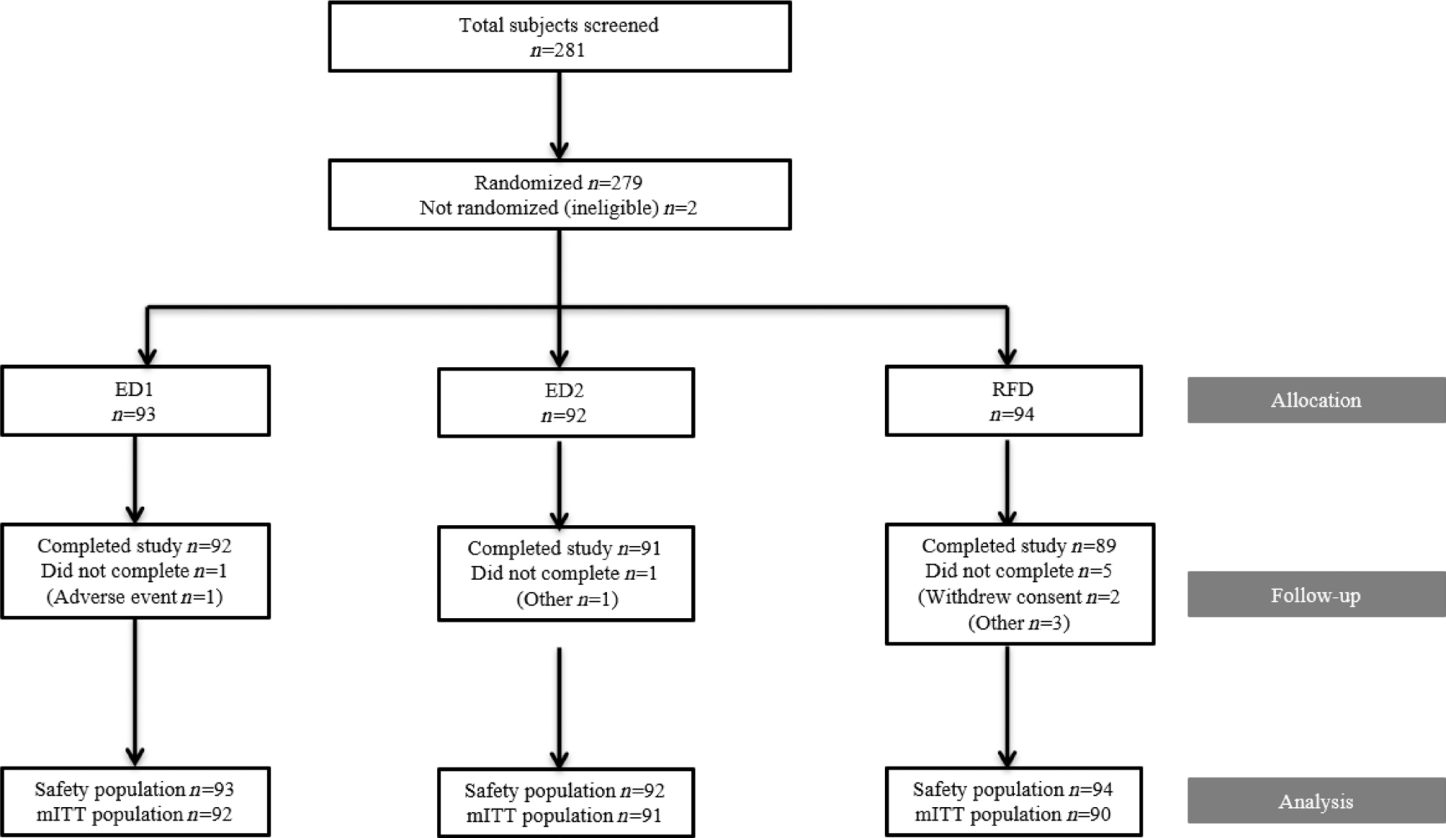
Subject disposition. mITT, modified intent‐to‐treat.

Two hundred and eighty‐one subjects were screened, 280 subjects were enrolled, 279 subjects were randomized (93 subjects in ED1, 92 subjects in ED2, and 94 in the RFD groups), and 272 subjects completed the study (Figure 1). Seven subjects did not complete the study: one subject due to AE (gingival swelling) (ED1), 4 subjects due to “other related to schedule issues” (1 for ED2 and 3 for RDF), and two subjects due to withdrawn consent (RDF). The first subjects enrolled in the study on September 5, 2022; the last subjects completed the study on December 4, 2022. All 279 randomized subjects were included in the safety population, and 273 of these were included in the mITT and Per Protocol populations (92 [98.9%] subjects in the ED1 group, 91 [98.9%] subjects in the ED2 group, and 90 [95.7%] subjects in the RFD group). A total of 52 (18.6%) subjects were included in both the MLSI Repeatability and VITA Repeatability Populations (12 [12.9%] subjects in the Experimental Dentifrice 1 group, 19 [20.7%] subjects in the ED2 group, and 21 [22.3%] in the RFD group). One hundred and sixty one (59.0%) of the subjects included in the mITT population were female, and the mean age of the mITT population was 41.1 years (range 18–65 years). Of the 279 subjects randomized, 279 subjects (100%) had a baseline overall MLSI (A × I) score ≥ 45 (high) and 39 subjects (14.3%) were smokers (Table 1). Baseline levels of stain were comparable between treatment groups. The demographic characteristics of the safety population were comparable between treatment groups (Supporting Information Table S1).

**TABLE 1:** Summary of baseline characteristics (mITT Population).

	Treatment group		
Characteristic	ED1 (*n* = 92)	ED2 (*n* = 91)	RFD (*n* = 90)
Sex, *n* (%)			
Male	40 (43.5)	41 (45.1)	31 (34.4)
Female	52 (56.5)	50 (54.9)	59 (65.6)
Age, years			
Mean	43.4	39.5	40.5
Range	18–65	19–65	18–65
Race, *n* (%)			
African American/African Heritage	7 (7.6)	5 (5.5)	6 (6.7)
American Indian or Alaskan Native	0	0	0
Asian–Central/South Asian Heritage	2 (2.2)	1 (1.1)	3 (3.3)
Asian–East Asian Heritage	1 (1.1)	1 (1.1)	1 (1.1)
Asian–Japanese Heritage	0	0	0
Asian–South East Asian Heritage	0	0	0
Native Hawaiian/Other Pacific Islander White–Arabic/North African Heritage White–White/Caucasian/European Heritage	0 1 (1.1) 81 (88.0)	0 1 (1.1) 91 (91.2)	0 1 (1.1) 79 (87.38)
Ethnicity, *n* (%)			
Hispanic or Latino	10 (10.9)	7 (7.7)	10 (11.1)
Not Hispanic or Latino	82 (89.1)	84 (92.3)	80 (88.9)
Stratification, *n* (%)			
Baseline total MLSI (A × I) < 45, smoker	0	0	0
Baseline total MLSI (A × I) < 45, non‐smoker	0	0	0
Baseline total MLSI (A × I) > = 45, smoker	13 (14.1)	13 (14.3)	13 (14.4)
Baseline total MLSI (A × I) > = 45, non‐smoker	79 (85.9)	78 (85.7)	77 (85.6)

### 3.2 Efficacy

**FIGURE 2:**
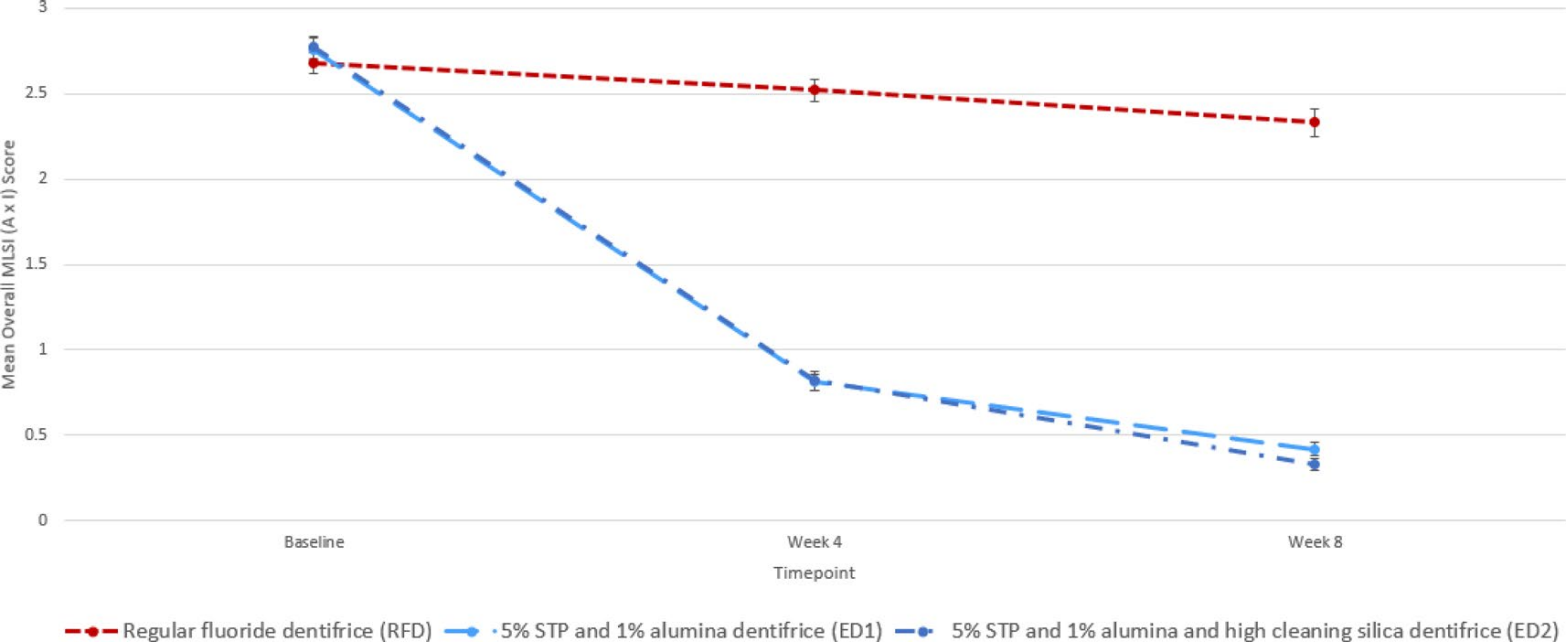
Mean all‐site MLSI (area (A) × intensity (I)) scores (mITT) over time for the three study dentifrices. MLSI, Macpherson modification of the Lobene Stain Index.

MLSI: The change from the baseline in the mean total MLSI (A × I) score (mean ± SD, (median)) at Week 8 was −2.34 ± 0.496 (−2.32) in the ED1 group, −2.44 ± 0.547 (−2.42) in the ED2 group, and −0.35 ± 0.674 (−0.26) in the RDF group. There was a statistically significant reduction in extrinsic dental stain as measured by the mean overall (all sites), body, gingival, and interproximal (combination of the distal and mesial areas) MLSI (A × I) score in all the treatment groups after 4 and 8 weeks of twice‐daily use of study products, compared to baseline (Table 2; Figure 2). For overall, gingival, interproximal, and body sites, statistically significant (*p* < 0.0001) between‐treatment differences favoring the experimental dentifrices (ED1 and ED2) compared to the RDF were observed at Weeks 4 and 8 (Table 3).

**TABLE 2:** Change from baseline in mean overall, gingival, and interproximal MLSI (area × intensity) score (mITT; *N* = 273).

	Treatment group					
Site/timepoint	ED1 (*n* = 92)		ED2 (*n* = 91)		RFD (*n* = 90)	
Total (overall)	Adj. mean (SE)	*p* (95% CI)	Adj. mean (SE)	*p* (95% CI)	Adj. mean (SE)	*p* (95% CI)
Week 4	−1.94 (0.046)	< 0.001 (−2.03, −1.85)	−1.93 (0.046)	< 0.001 (−2.02, −1.84)	−0.19 (0.047)	< 0.001 (−0.28, −0.10)
Week 8	−2.33 (0.048)	< 0.001 (−2.42, −2.23)	−2.42 (0.049)	< 0.001 (−2.52, −2.32)	−0.38 (0.049)	< 0.001 (−0.48, −0.28)
Gingival						
Week 4	−2.39 (0.064)	< 0.001 (−2.52, −2.27)	−2.40 (0.065)	< 0.001 (−2.52, −2.27)	−0.17 (0.065)	< 0.001 (−0.30, 0.04)
Week 8	−2.96 (0.067)	< 0.001 (−3.09, −2.83)	−3.09 (0.067)	< 0.001 (−3.22, −2.96)	−0.46 (0.068)	< 0.001 (−0.60, −0.33)
Interproximal						
Week 4	−2.27 (0.055)	< 0.001 (−2.37, −2.6)	−2.25 (0.056)	< 0.001 (−2.34, −2.14)	−0.20 (0.056)	0.0003 (−0.31, −0.09)
Week 8	−2.69 (0.057)	< 0.001 (−2.81, −2.58)	−2.81 (0.057)	< 0.001 (−2.92, −2.70)	−0.40 (0.058)	< 0.001 (−0.52, −0.29)
Body						
Week 4	−0.82 (0.046)	< 0.001 (−0.91, −0.73)	−0.84 (0.047)	< 0.001 (−0.93, −0.75)	−0.20 (0.047)	< 0.001 (−0.29, −0.10)
Week 8	−0.95 (0.043)	< 0.001 (−1.04, −0.87)	−0.96 (0.043)	< 0.001 (−1.04, −0.87)	−0.26 (0.043)	< 0.001 (−0.35, −0.18)

Abbreviations: CI, confidence interval; MLSI, Macpherson modification of the Lobene Stain Index; SE, standard error.

**TABLE 3:** Statistical comparison of ED1 and ED2 to RFD for mean overall, gingival, and interproximal MLSI (area × intensity) score (mITT; N = 273).

	Treatment group			
Site/timepoint	ED1 (*n* = 92)		ED2 (*n* = 91)	
Total (overall)	Adj. mean difference (SE)	*p* (95% CI)	Adj. mean difference (SE)	*p*lue
Week 4	−1.75 (0.066)	< 0.001 (−1.88, −1.62)	−1.74 (0.066)	< 0.001 (−1.87, −1.61)
Week 8	−1.95 (0.069)	< 0.001 (−2.08, −1.81)	−2.04 (0.069)	< 0.001 (−2.18, −1.90)
Gingival				
Week 4	−2.23 (0.092)	< 0.001 (−2.41, −2.05)	−2.23 (0.092)	< 0.001 (−2.41, −2.05)
Week 8	−2.50 (0.095)	< 0.001 (−2.69, −2.31)	−2.63 (0.095)	< 0.001 (−2.81, −2.44)
Interproximal				
Week 4	−2.06 (0.079)	< 0.001 (−2.22, −1.91)	−2.04 (0.079)	< 0.001 (−2.20, −1.89)
Week 8	−2.29 (0.081)	< 0.001 (−2.45, −2.13)	−2.41 (0.082)	< 0.001 (−2.57, −2.25)
Body				
Week 4	−0.63 (0.066)	< 0.001 (−0.76, −0.50)	−0.64 (0.066)	< 0.001 (−0.77, −0.51)
Week 8	−0.69 (0.061)	< 0.001 (−0.81, −0.57)	−0.69 (0.061)	< 0.001 (−0.81, −0.57)

**FIGURE 3:**
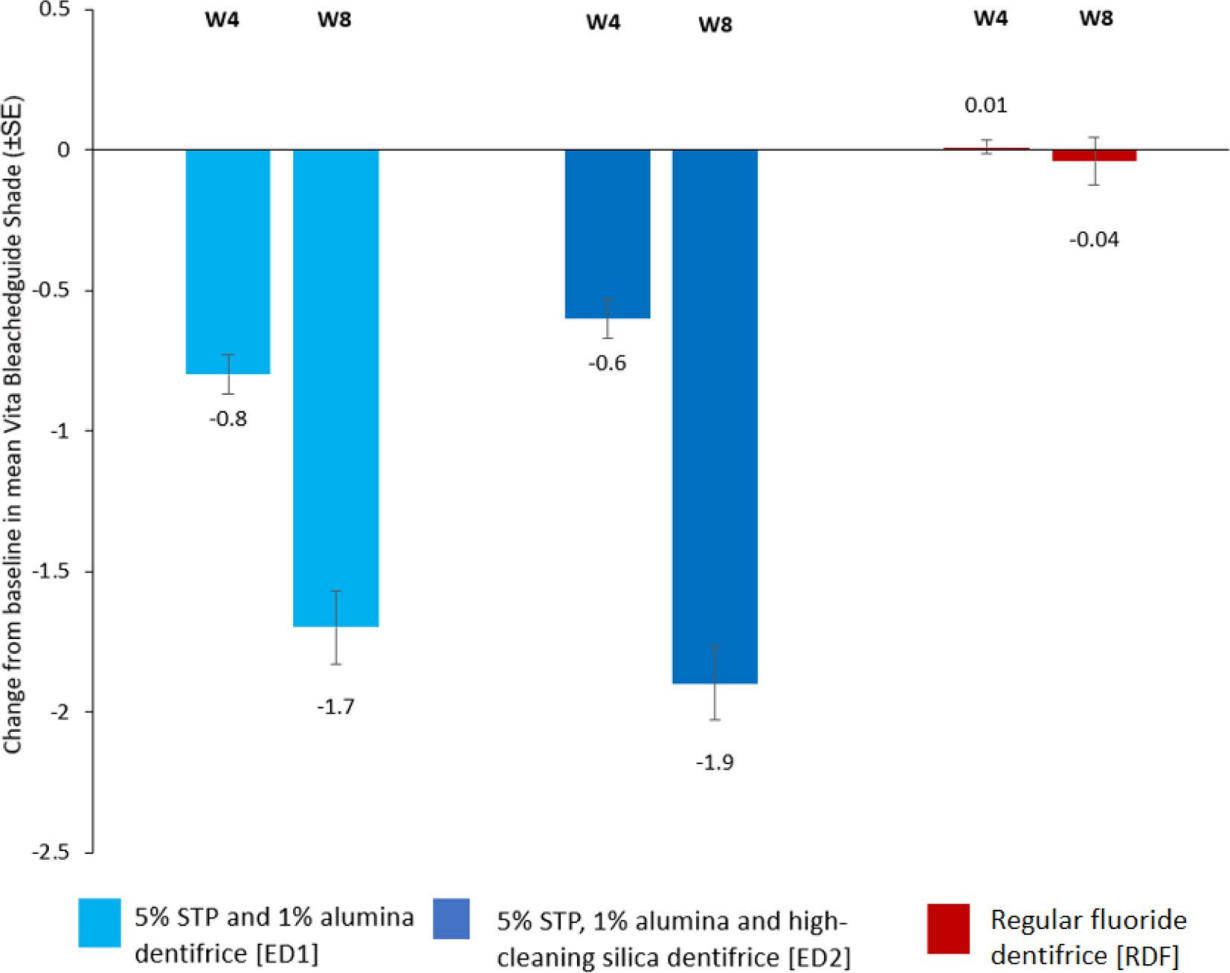
VITA shade scores (mITT) change from baseline for the three study dentifrices.

VITA Score: The change from Baseline in the mean VITA shade score (mean ± SD, (median)) at Week 8 was −1.72 ± 1.419 (−2.00) in the Experimental Dentifrice 1 group, −1.86 ± 1.498 (−2.00) in the Experimental Dentifrice 2 group, and −0.04 ± 0.812 (0.00) in the reference dentifrice group. There was a significant reduction (improvement) in VITA tooth shade score, as measured by examiner assessment against the VITA Bleachedguide in the Experimental dentifrice groups after 4 and 8 weeks of twice‐daily use compared to baseline. No significant change in tooth shade score, compared to baseline was observed for the reference dentifrice at either time point (Figure 3, Table 4). Statistically significant (*p* < 0.0001) between‐treatment differences favoring the experimental dentifrices (ED1 and ED2) compared to the reference dentifrice were observed in the mean VITA shade score at Weeks 4 and 8 (Table 4).

**TABLE 4:** Change from baseline and statistical comparison of ED1 and ED2 to RFD for VITA shade score (mITT; *N* = 273).

	Treatment group					
Site/timepoint	ED1 (*n* = 92)		ED2 (*n* = 91)		RFD (*n* = 90)	
VITA score	Adj. mean (SE)	*p* (95% CI)	Adj. mean (SE)	*p* (95% CI)	Adj. mean (SE)	*p* (95% CI)
Week 4	−0.81 (0.068)	< 0.001 (−0.94, −0.67)	−0.59 (0.068)	< 0.001 (−0.73, −0.46)	0.01 (0.069)	0.929 (−0.13, 0.14)
Week 8	−1.73 (0.134)	< 0.001 (−1.99, −1.46)	−1.85 (0.134)	< 0.001 (−2.12, −1.59)	−0.04 (0.136)	0.754 (−0.31, 0.22)
Comparison with SDF						
Week 4	−0.81 (0.097)	< 0.001 (−1.00, −0.62)	−0.60 (0.097)	< 0.001 (−0.79, −0.41)		
Week 8	−1.68 (0.190)	< 0.001 (−2.06, −1.31)	−1.81 (0.191)	< 0.001 (−2.19, −1.44)		

Repeatability: The repeatability analysis demonstrated *κ* = 0.9924, 0.9865, and 0.9970 for MLSI Area, MLSI Intensity, and Vitashade assessments respectively. These *κ* values therefore established excellent intra‐examiner agreement for all efficacy assessments (*κ* > 0.98).

### 3.3 Safety

A total of 22 treatment‐emergent AEs (TEAEs) were reported in 16 subjects. Of these, 12 were oral AEs reported in nine subjects. 10 of the oral AEs were deemed treatment‐related: one case of gingival pain and one case of gingival swelling (ED1 treatment group); one of each of angular cheilitis, chapped lips, erythema, dry lip, lip exfoliation, lip pain, oral mucosal exfoliation, and skin fissures (ED2 treatment group). There were no treatment‐related treatment‐emergent AEs in the RFD group. All treatment‐related AEs were mild in intensity. In the case of gingival swelling, the subject was discontinued from the study and the event resolved with sequelae 1 day after discontinuing treatment. All other treatment‐emergent AEs resolved on study completion. No serious AEs were reported. The treatment‐related TEAEs are consistent with other studies investigating tooth‐whitening dentifrice formulations.

## 4 Discussion

There is considerable demand for oral hygiene products that bleach tooth stains (intrinsic and extrinsic), but also those that whiten teeth by reducing and preventing extrinsic dental stains. In 1948, Kitchin and Robinson [7] wrote that “One should use only as much abrasion as necessary to clean one's teeth.” Since this time, special dental‐grade abrasives have been developed with the aim of delivering effective stain removal with lower abrasivity, and the introduction of chemical cleaning agents has permitted lower levels of abrasive to be employed, while still maintaining effective stain removal. Most recently, dentifrices with chemical bleaching agents (low levels of hydrogen peroxide or peroxide precursors) have been introduced to the market that purport to bleach tooth stains rather than chemically or physically remove them. However, the visual perception of tooth color improvement is multifactorial and is more complex than just surface stain (accumulation, removal, or bleaching). The interaction of the abrasive and any chemical cleaning/bleaching agent on the physical parameters of the enamel structure/surface (reflection characteristics) contributes to the visual tooth color [21].

The physical stain‐removal ingredients generally found in whitening dentifrices are abrasives such as silica, calcium carbonate, charcoal, and more recently specific fine grades of alumina. With the action of tooth brushing, the abrasive particles remove the surface‐bound stain [5, 6, 7, 8, 9, 14, 15], but in doing so they can also confer a degree of abrasion (and or polishing) to the dental hard tissues resulting in dental wear over time [5, 8, 9, 22, 23]. Abrasive wear can be minimized by reducing the quantity of abrasive in the dentifrice formulation; usually at the cost of reducing stain control efficiency.

The desired goal of whitening dentifrices is to obtain a level of stain removal or whitening efficiency that satisfies consumer needs while minimizing abrasive wear. This is especially important for people at risk of erosive toothwear or experiencing dentin hypersensitivity who would benefit most from low abrasion dentifrices. Two previous studies reported in the literature have explored the stain control properties of low abrasion 5% STP/1% micronized alumina dentifrices (with 5% KNO_3_), designed for people at risk of erosive toothwear and/or with dentin hypersensitivity. In an exploratory stain‐removal study, Young et al. [14] reported statistically significant reductions in extrinsic stain (as measured by MLSI) for all study dentifrices; however, no significant differences or trends between study products were observed (albeit the study was not powered to detect between‐treatment differences). Milleman et al. [15] reported on the potential benefits of combining the physical cleaning properties of micronized alumina with the chemical cleaning properties of STP, to provide effective stain prevention; a 5% STP, 1% alumina low abrasion dentifrice (RDA 40) demonstrated the lowest stain score [greater stain prevention] (as measured by MLSI) and statistically significantly less stain compared to an STP alone dentifrice (RDA 13), a regular dentifrice (RDA 80) and a higher RDA whitening dentifrice (RDA 142), following 8 weeks of twice‐daily use.

This randomized controlled clinical trial evaluated the stain removal and for the first time whitening (tooth shade) efficacy of two experimental dentifrices containing micronized alumina (RDA 40) or a combination of alumina and silica (RDA 120), together with the chemical cleaning agent STP, compared with a regular abrasive dentifrice (RDA ~ 90). This study employed both a standardized stain index measure (MSLI) and a visual assessment of shade change so that a complete understanding of both the stain‐removal and shade‐improvement properties of the experimental dentifrices could be evaluated.

The results of this study are in agreement with studies reported in the literature on the stain removal and long‐term stain prevention properties of low abrasion, high‐cleaning dentifrices with physical stain removal (1% micronized alumina), and chemical cleaning (5% STP) [14, 15]. These formulations offer effective stain removal and whitening while minimizing wear. Seong et al. [23], employing an in situ abrasion model, reported that brushing with a 5% STP/1% micronized alumina dentifrice resulted in significantly less dentin loss compared with a higher abrasivity daily‐use whitening dentifrice. In this study, it is interesting to observe that the addition of high‐cleaning abrasive silica to the 1% micronized alumina/5% STP formulations, did not offer a meaningful enhancement in stain removal or tooth shade beyond that demonstrated by the 1% micronized alumina/5% STP system alone. Such an observation was unexpected as it does not follow the cleaning/abrasion paradigm. However, the stain‐removal potential of 5%STP/1% micronized alumina alone was observed to be highly efficient, removing almost all the extrinsic stain (as evidenced by a mean MSLI (A x I) of < 0.5) following 8 weeks of twice‐daily use, such that there was potentially limited scope for further stain removal.

Previous studies evaluating the efficacy of 1% micronized alumina and 5% STP‐containing dentifrices have only investigated the stain‐removal and/or stain‐prevention efficacy using clinical stain indices such as MLSI. To the best of the authors' knowledge, the efficacy of micronized alumina/STP‐based dentifrices to improve tooth shade has not been explored previously. Therefore, the secondary objective of this study was to explore the improvements in tooth shade (as measured by examiner assessment using the VITA Bleachedguide) following 4 and 8 weeks of twice‐daily brushing with the experimental dentifrices. At all time points, significant and meaningful improvements in VITA tooth shade were observed for the experimental dentifrices compared to the baseline. The change in shade score (for ED1 and ED2) ranged from 3 shades darker to 7.5 shades lighter, with a median value for both experimental dentifrices of 2 shades lighter compared to the baseline. No change in shade score was observed for the regular dentifrice [RFD median 0.0 (2.0 to −4.0)].

The VITA shade guide selected for use in this study (VITA Bleachedguide 3D‐MASTER) has the advantage over other shade guides in providing a linear presentation of tooth shade, which enables a more accurate assessment of shade changes [24]. Furthermore, it is specified as suitable by the American Dental Association for use in determining the efficacy of tooth‐whitening products [25]. The Bleachedguide has been previously utilized in many studies evaluating the efficacy of tooth‐whitening treatments [26, 27, 28]. A study evaluating the shade tabs of the Bleachedguide found all observers were correctly able to rank all of the shade tabs in the correct order (lightest to darkest) 100% of the time [24], demonstrating that the shade difference between adjacent shade tabs is easily distinguishable. Additionally, a study of 80 independent observers on denture teeth found that the perceptibility threshold where 50% of observers could successfully determine shade difference corresponded to a ΔE value of 1.9 (95% CI of 1.7–2.1) [29]. The mean difference in the shade of adjacent tabs in the Bleachedguide has been spectrophotometrically measured as ΔE = 3.0 [29], demonstrating that the shade separation between tabs is meaningful. Taken together, these findings demonstrate that the median change in tooth shade of 2.0 observed in this study is both visually distinguishable and meaningful.

The effectiveness of dentifrices to whiten teeth by extrinsic stain removal can be evaluated by several methods, from exploring the removal of induced stain (forced), prevention of induced stain build‐up (forced) [30, 31] to remove natural stains or prevent natural stain build‐up over various time periods [14, 15]. This study employed a clinical model that investigated the removal of natural stains, in volunteers with a moderate level of dental stain over 8 weeks of twice‐daily use. The model and duration of the study were chosen to reflect the level of stain/tooth shade considered representative of people seeking a whitening benefit from daily use of whitening dentifrice. A limitation of this study is that the results may not necessarily be generalizable to people with a high propensity to stain build‐up, for example through atypical dietary, environmental, or medicinal influences. Another limitation was that the study was relatively short. While the study length conformed to ADA guidelines [32], it is not possible to comment on the maximum shade improvement possible as a plateau was not reached. Future studies should explore participants with a wider range of tooth stains at screening and consider extending the evaluation period to establish the maximum achievable performance.

## 5 Conclusion

This clinical study demonstrates that a dentifrice with 1% micronized alumina and 5% STP can provide large and clinically meaningful superior reductions in tooth stain (MLSI) compared to a regular dentifrice, which translates into a two‐shade improvement in median tooth shade while maintaining low abrasion.

## Author Contributions

C.R.P., G.R.B., and G.S. contributed to the design, conduct, and reporting of the study. M.P., J.G., and J.Q. were involved in the conduct of the study. All authors had access to the final study report, made contributions to the development of the manuscript, had final responsibility for the decision to submit, and approved the submitted version.

## Conflicts of Interest

C Parkinson, G Burnett, G Smith and M Pradhan report that they are employees of Haleon, a manufacturer of products evaluated in this study. J. Gallob and J. Qaqish report that they received funding from Haleon to conduct this study.

## Supporting information


Data S1.

## Data Availability

The data that support the findings of this study are openly available in Haleon study register at https://haleon‐study‐register.idea‐point.com/StudyRegister.aspx, reference number NCT06150573.
