# Supplementary material for: A Randomized Clinical Study Investigating the Stain Removal Efficacy of Two Experimental Dentifrices
Source: J Esthet Restor Dent. 2025 Jan 13;37(6):1483–93. doi: 10.1111/jerd.13373 (PMC12087941; doi:10.1111/jerd.13373)
Supplement: Supplementary file 1 — Data S1. [file JERD-37-1483-s001.docx]

Supplemental Data:

**Table I** Summary of Baseline Characteristics (Safety Population)

| **Characteristic** | **Treatment group** | | | | | | |  |
| --- | --- | --- | --- | --- | --- | --- | --- | --- |
|  | **ED1 (n=93)** | | **ED2 (n=92)** | | **RFD (n=94)** | | |  |
| **Sex, n (%)** | |  | |  | |  | | |
| Male | | 40 (43.0) | | 41 (44.6) | | 34 (36.2) | | |
| Female | | 53 (57.0) | | 51 (55.4) | | 60 (63.8) | | |
| **Age, years** | |  | |  | |  | | |
| Mean | | 43.4 | | 39.8 | | 40.4 | | |
| Range | | 18–65 | | 19–65 | | 18–65 | | |
| **Race, n (%)** | |  | |  | |  |  |  |
| African American/African Heritage | | 7 (7.5) | | 5 (5.4) | | 6 (6.4) | | |
| American Indian or Alaskan Native | | 0 | | 0 | | 0 | | |
| Asian - Central/South Asian Heritage | | 2 (2.2) | | 1 (1.1) | | 3 (3.2) | | |
| Asian - East Asian Heritage | | 1 (1.1) | | 1 (1.1) | | 1 (1.1) | | |
| Asian - Japanese Heritage | | 0 | | 0 | | 0 | | |
| Asian - South East Asian Heritage | | 0 | | 0 | | 0 | | |
| Native Hawaiian/Other Pacific Islander  White - Arabic/North African Heritage  White - White/Caucasian/European Heritage | | 0  1 (1.1)  82 (88.2) | | 0  1 (1.1)  84 (91.3) | | 0  1 (1.1)  83 (88.3) | | |
| **Ethnicity, n (%)** | |  | |  | |  | | |
| Hispanic or Latino | | 10 (10.8) | | 7 (7.6) | | 10 (10.6) | | |
| Not Hispanic or Latino | | 83 (89.2) | | 85 (92.4) | | 84 (89.4) | | |
